# Interleukin-1 blockade overcomes erlotinib resistance in head and neck squamous cell carcinoma

**DOI:** 10.18632/oncotarget.12590

**Published:** 2016-10-12

**Authors:** Aditya Stanam, Katherine N. Gibson-Corley, Laurie Love-Homan, Nnamdi Ihejirika, Andrean L. Simons

**Affiliations:** ^1^ Interdisciplinary Human Toxicology Program, The University of Iowa, Iowa City, IA, USA; ^2^ Department of Pathology, The University of Iowa, Iowa City, IA, USA; ^3^ Lincoln University of the Commonwealth of Pennsylvania, Lincoln, PA, USA; ^4^ Department of Radiation Oncology, The University of Iowa, Iowa City, Iowa City, IA, USA; ^5^ Holden Comprehensive Cancer Center, The University of Iowa, Iowa City, IA, USA; ^6^ Roy J. and Lucille A. Carver College of Medicine, The University of Iowa, Iowa City, IA, USA

**Keywords:** interleukin-1, erlotinib resistance, interleukin-1 receptor antagonist, anakinra, head and neck squamous cell carcinoma

## Abstract

Erlotinib has demonstrated poor clinical response rates for head and neck squamous cell carcinoma (HNSCC) to date and the majority of respondents acquire resistance to erlotinib relatively quickly. To elucidate novel pathways involved in erlotinib resistance, we compared the gene expression profiles of erlotinib-resistant (ER) vs. erlotinib-sensitive (ES) HNSCC cell lines. Enrichment analysis of microarray data revealed a deregulation of the IL-1 signaling pathway in ER versus ES-HNSCC cells. Gene expression of interleukin-1 alpha (IL1A) and interleukin-1 beta (IL1B) were significantly upregulated by > 2 fold in ER-SQ20B and ER-CAL 27 cells compared to their respective ES-cells. Secretion of the IL-1 receptor antagonist (IL-1RA) was significantly reduced in ER-cells compared to ES-cells. Blockade of IL-1 signaling using a recombinant IL-1R antagonist (anakinra) was able to inhibit the growth of ER-SQ20B and ER-CAL 27 but not ES-SQ20B and ES-CAL 27 xenografts as a single agent and in combination with erlotinib. ER-SQ20B xenografts treated with anakinra ± erlotinib were found to be less vascularized than ER-SQ20B xenografts treated with water or erlotinib. Mice bearing ER-SQ20B xenografts had significantly lesser circulating levels of G-CSF and IL-1β when treated with anakinra ± erlotinib compared to those treated with water or erlotinib alone. Furthermore, augmented mRNA levels of IL1A or interleukin-1 receptor accessory protein (IL1RAP) were associated with shortened survival in HNSCC patients. Altogether, blockade of the IL-1 pathway using anakinra overcame erlotinib resistance in HNSCC xenografts and may represent a novel strategy to overcome EGFR inhibitor resistance for treatment of HNSCC patients.

## INTRODUCTION

Epidermal growth factor receptor (EGFR) is an important molecular target for antineoplastic therapy in head and neck squamous cell carcinoma (HNSCC) as it is found to be upregulated and overexpressed in the majority of HNSCCs and is associated with a poor clinical prognosis [1, 2, 3]. Although the FDA approved EGFR monoclonal antibody cetuximab demonstrates some clinical activity, this approach has shown limited success due to unexplained poor tumor responses and the rapid development of drug resistance [4, 5]. Moreover, EGFR tyrosine kinase inhibitors (TKIs) such as erlotinib and gefitinib have demonstrated poor clinical response rates (5-10%) and thus disappointing results in clinical trials for HNSCC to date [6, 7].

In contrast to HNSCC, EGFR TKIs induce significant tumor regression in NSCLC patients, but only in patients that harbor somatic mutations in exons of EGFR that code for the tyrosine kinase domain (~15-35% of NSCLC patients) [8, 9]. However, these particular NSCLC patients still eventually acquire resistance to EGFR inhibitors despite their sensitizing EGFR mutation status [8, 9]. Additionally, these particular EGFR-sensitizing mutations are rarely observed in HNSCC tumors.

Numerous studies have proposed mechanisms that may be responsible for poor responses and treatment failures due to treatment with EGFR-TKIs [10–13]. The most well-known mechanisms include acquisition of a secondary EGFR mutation (most commonly T790M in exon 20), KRAS mutations and amplification of the MET oncogene [14, 15], but in most patients, the mechanisms of resistance are unknown. More importantly, the above described resistance mechanisms are relevant to NSCLC tumors and are rare or not applicable to HNSCC tumors. Given the number of ongoing clinical trials testing the use of EGFR TKIs in HNSCC patients, there is a greater need to understand the mechanisms of resistance to EGFR TKIs in order to improve the efficacy of these agents, to develop optimal combinatorial therapies, and to select patients (based on biomarkers) who will likely benefit from EGFR TKIs.

Previous studies in our laboratory uncovered a novel link between HNSCC tumor response to the EGFR TKI erlotinib and interleukin-1 (IL-1)-mediated inflammation [16]. We observed that erlotinib treatment activated the IL-1 pathway in HNSCC cells which limited the efficacy of erlotinib in HNSCC cells [16]. The IL-1 pathway plays a central role in inflammatory responses by regulating the expression of various inflammatory genes in immune cells. IL-1 signaling is activated when either of the agonistic IL-1 ligands i.e. IL-1α or IL-1β binds to the IL-1 receptor 1 (IL-1R1) which then forms a complex with the IL-1 receptor accessory protein (IL-1RAcP). This heterodimeric complex recruits an adapter protein myeloid differentiation primary response gene 88 (MyD88) which recruits IL-1 receptor-associated kinases (IRAK4, IRAK1, and IRAK2) and TRAF6 [17, 18]. These signaling events are important for the assembly of a complex containing MAP3K7 (Tak1) which can activate NF-κB and MAPK signaling leading to the expression of IL-1 target genes. Negative regulation of the IL-1 pathway includes the IL-1 receptor antagonist (IL-1Ra), which competitively binds to IL-1R1 and prevents the binding of the agonistic IL-1 ligands, and IL-1R2 which lacks a signal transduction domain and serves as a decoy receptor [17].

IL-1 signaling is also associated with tumor growth, angiogenesis, metastasis, and cancer-associated wasting syndrome (cachexia) [18, 19]. IL-1 pathway signaling activates pathways leading to the expression of numerous pro-inflammatory cytokines involved in tumor survival and the infiltration of various immune cells to the tumor, which further increases the inflammatory and pro-survival microenvironment of the tumor [20, 21]. Because of this, the IL-1 pathway is seen as a potent inducer of inflammation by activating and reinforcing a vicious cycle of pro-inflammatory cytokine release which may promote and sustain tumor survival leading to poor drug response and drug resistance.

Here we show that interleukin-1 (IL-1) signaling is upregulated in erlotinib-resistant HNSCC cells compared to their erlotinib-sensitive parental cells and that blockade of IL-1 signaling with anakinra is sufficient to overcome erlotinib resistance in HNSCC cells. Our results strongly suggest that the IL-1 pathway may serve as a novel mechanism responsible for tumor resistance to EGFR TKIs in HNSCC therapy.

## RESULTS

### A pro-inflammatory gene signature is associated with erlotinib resistance

**Figure 1:**
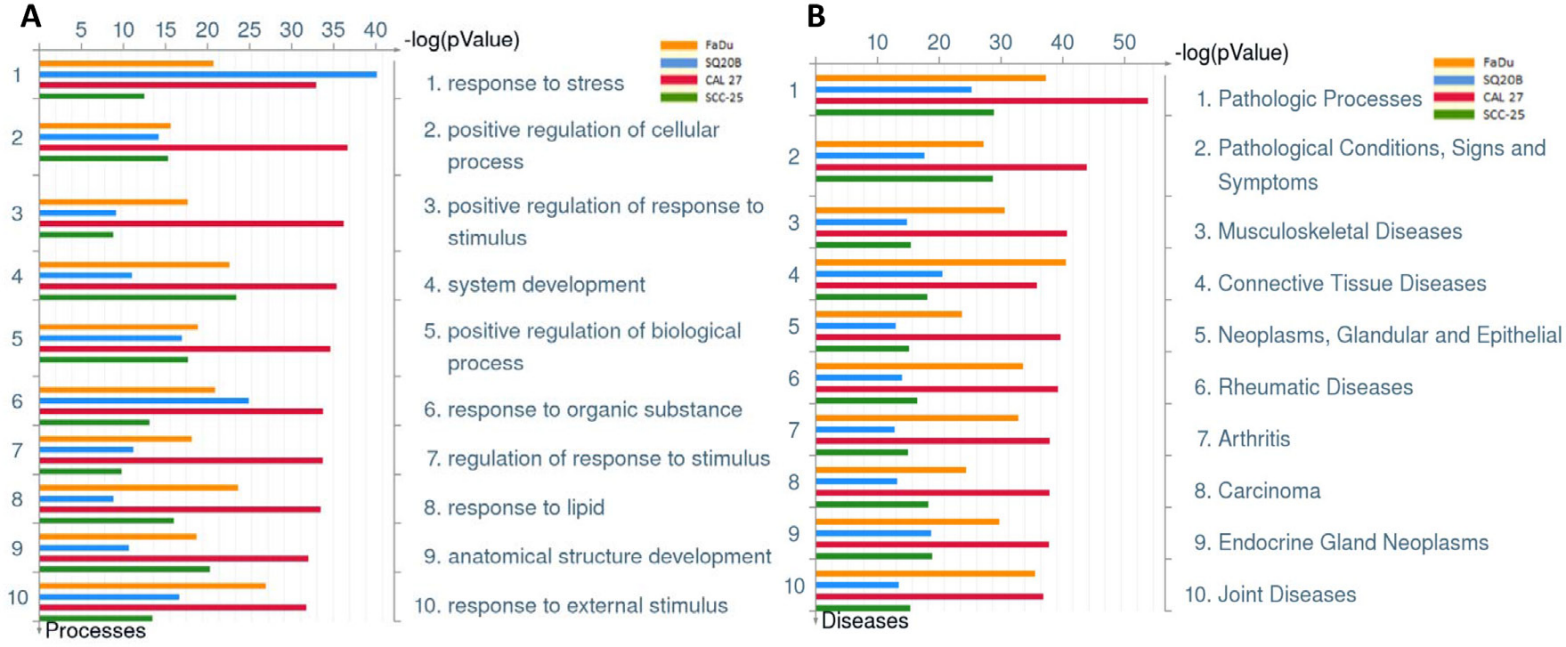
Gene Ontology Processes and Diseases (by biomarkers) associated with erlotinib-resistance in HNSCC cells Shown are the top ten deregulated biological processes **A.**, and top ten deregulated diseases (by biomarkers) **B.** from differentially regulated transcripts comparing microarray data from erlotinib-resistant FaDu (yellow bars), SQ20B (blue bars), CAL 27 (green bars), and SCC-25 (red bars) HNSCC cells as compared to their respective erlotinib-sensitive HNSCC cells.

**Figure 2:**
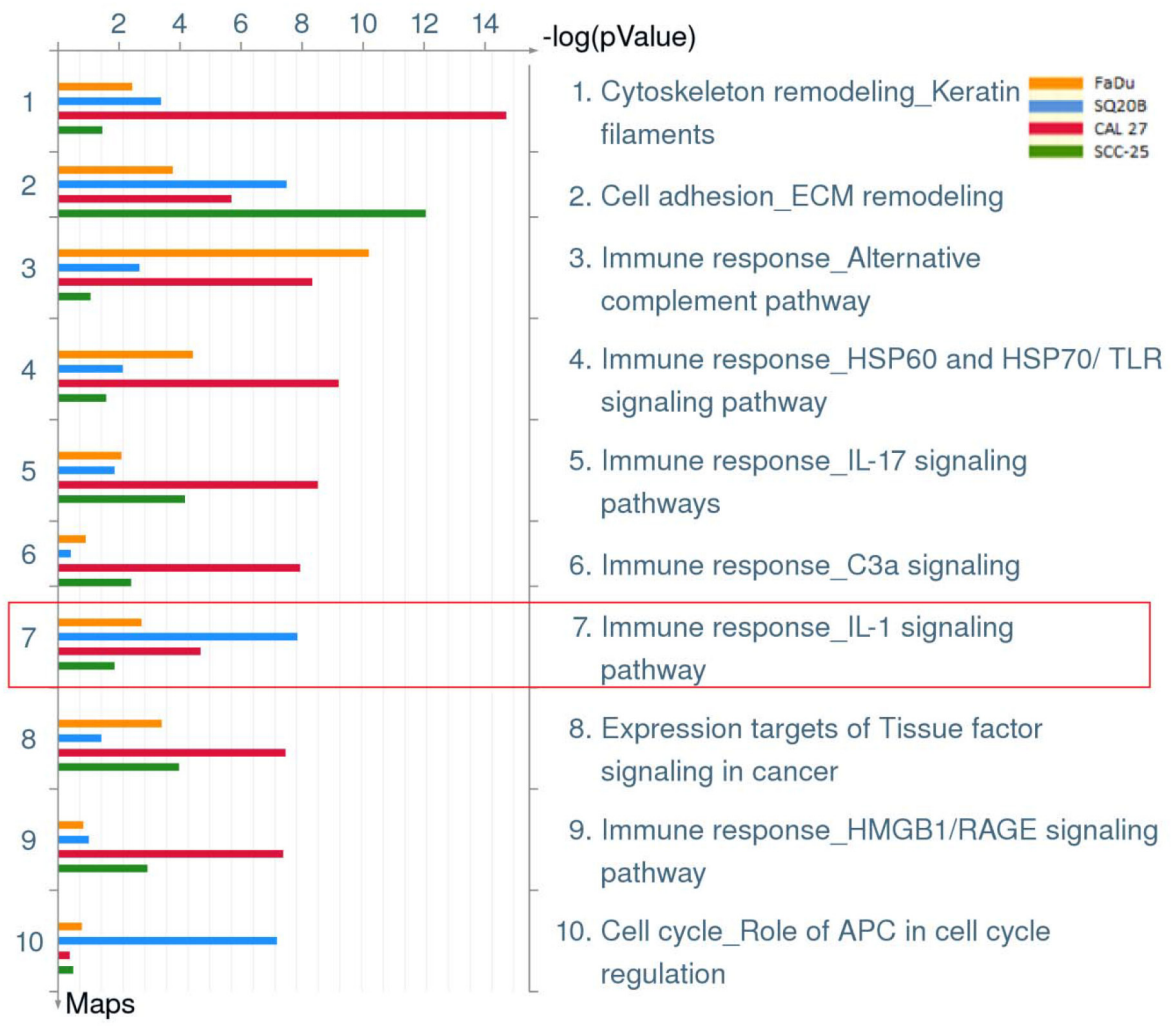
Biological pathways deregulated in erlotinib resistant HNSCC cells Shown are the top ten deregulated pathways from differentially regulated transcripts comparing microarray data from erlotinib-resistant FaDu (yellow bars), SQ20B (blue bars), CAL 27 (green bars), and SCC-25 (red bars) HNSCC cells as compared to their respective erlotinib-sensitive HNSCC cells. Enclosed in red box is pathway #7 which represents the IL-1 signaling pathway.

To investigate if an increased pro-inflammatory gene signature mediated by deregulated IL-1 signaling was involved in erlotinib resistance in HNSCC cells, gene expression analyses were performed on erlotinib-resistant (ER) and parental erlotinib-sensitive (ES) FaDu, CAL 27, SQ20B and SCC-25 HNSCC cells. The development and characterization of these ER-HNSCC cells have been previously described [22] and the raw gene expression data has been reported previously [GEO accession #GSE62061]. Combined enrichment analysis of all 4 ER-HNSCC cells compared to their respective ES counterparts revealed that the majority of the top ten deregulated Gene Ontology (GO) processes associated with ER-HNSCC cells are related to stress or stimulus responses (Figure 1A). The top ten significant diseases that were identified in ER-HNSCC cell lines were expected conditions such as ‘Neoplasms, Glandular and Epithelial’, ‘Carcinoma’, ‘Endocrine Gland Neoplasms’ and non-specific conditions such as ‘Pathologic Processes’ and ‘Pathological Conditions, Signs and Symptoms’ (Figure 1B). However, systemic inflammatory disorders were also identified in the gene signature from ER-HNSCC cells such as ‘Arthritis’, ‘Rheumatic Diseases’, ‘Joint Diseases’, ‘Connective Tissue Diseases’, and ‘Musculoskeletal Diseases’ (Figure 1B). The majority of the biological pathways deregulated in ER-HNSCC cells pertained to immune response pathways such as 'Alternative complement pathway', 'HSP60 and HSP70/TLR signaling pathway', 'IL-17 signaling pathway', ‘C3a signaling’, HMGB1/RAGE signaling pathway' and ‘IL-1 signaling pathway’(Figure 2) all of which are involved in pro-inflammatory signaling. Altogether, the gene expression analyses suggested that a pro-inflammatory gene signature may be involved in ER-HNSCC cell lines and that the IL-1 signaling pathway has been identified as one of the deregulated pathways in erlotinib resistance.

### Increased expression of IL-1 pathway genes is associated with erlotinib resistance

A closer look at the differential expression of IL-1 pathway genes that may be involved in erlotinib resistance showed that the majority of IL-1 pathway ligands, receptors, signaling members and target genes were significantly deregulated in ER-SQ20B and ER-CAL 27 cell lines compared to ER-FaDu and ER-SCC-25 cell lines (Table 1). Notably, both of the activating IL-1 pathway ligands *IL1A* and *IL1B* were significantly upregulated by greater than 2-fold in ER-SQ20B and ER-CAL 27 compared to their respective ES-cell lines (Table 1). Additionally, in ER-SQ20B, there were significant increases in gene expression of IL1R1, IL1R2, IRAK1 and MYD88 which all play a role in the IL-1 signaling cascade (Table 1). Altogether, these results support a possible role of IL-1 signaling in ER-HNSCC cell lines.

**Table 1:** Differential Expression of IL-1 Pathway Genes in Erlotinib-Resistant (ER) versus Erlotinib-Sensitive (ES) HNSCC cells

Gene	Gene description	Fold change (ER *vs.* ES)			
		FaDu	SQ20B	CAL 27	SCC-25
*IL1A*	Interleukin-1 alpha	−1.08	8.80*	8.36*	−2.04*
*IL1B*	Interleukin-1 beta	−1.92*	3.11*	2.87*	−2.12*
*IL1RN*	Interleukin-1 receptor antagonist	−2.08*	−1.09	−1.28	−2.11*
*IL1R1*	Interleukin-1 receptor 1	1.43	−2.53*	−1.11	1.74*
*IL1R2*	Interleukin-1 receptor 2	−1.07	−1.43*	−1.30	−1.10
*IL1RAP*	Interleukin-1 receptor accessory protein	−1.14	2.01*	1.16	−1.16
*IRAK1*	Interleukin-1 receptor-associated kinase 1	−1.03	−1.70*	−1.14	1.05
*IRAK4*	Interleukin-1 receptor-associated kinase 4	1.05	1.24	1.08	−1.13
*MYD88*	Myeloid differentiation primary response gene 88	1.13	2.13*	−1.59*	−1.36*
*TOLLIP*	Toll interacting protein	1.14	1.87*	1.05	−1.23
*TRAF6*	TNF receptor-associated factor 6	1.12	1.25	−1.06	1.07
*CCL2*	Chemokine (C-C Motif) ligand 2	1.29	1.28	59.56*	1.01
*DUSP1*	Dual specificity phosphatase 1	2.15*	5.47*	1.05	3.35*
*IL8*	Interleukin-8	4.76*	−1.52*	13.29*	1.23
*NFKBIA*	Nuclear factor of kappa light polypeptide gene enhancer in B-Cells inhibitor, alpha	1.79*	−1.79*	2.20*	1.17
*PTGS2*	Prostaglandin-endoperoxide synthase 2	−1.20	5.06*	6.99*	−1.05

* **indicates false discovery rate (FDR) significance < 0.05.**

### Increased expression of IL-1 pathway ligands may be associated with erlotinib resistance

**Figure 3:**
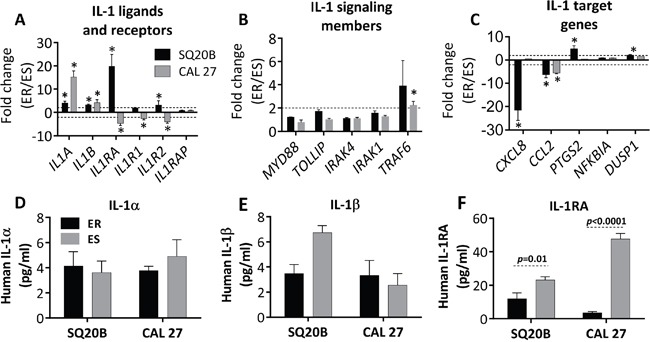
Validation of select IL-1 pathway genes in erlotinib resistant (ER) vs. erlotinib sensitive (ES) HNSCC cells Expression of IL-1 ligands and receptors **A.**, signaling genes **B.**, and select IL-1 target genes **C.** in ER- vs. ES- SQ20B and –CAL 27 cells were analyzed by quantitative RT-PCR. GAPDH or 18S was used as an endogenous control. Dotted horizontal lines indicate fold change of ±2. Secretion of IL-α **D.**, IL-1β **E.**, and IL-1RA **F.** in cell culture supernatants were analyzed by sandwich ELISA in ER- vs. ES- SQ20B and CAL 27 cells; and the concentrations were normalized to cell number. Fold change values were calculated by the anti-log of delta delta CT values (2^-delta delta CT values). * indicates significantly (fold change > +2 or < −2 and false discovery rate (FDR) < 0.05).

Because we saw the most significant activation of the IL-1 pathway in ER-SQ20B and ER-CAL 27 (Figure 2, Table 1), we continued our studies with these 2 cell lines. The IL-1 pathway ligands and receptors (Figure 3A), signaling members (Figure 3B) and target genes (Figure 3C) from Table 1 were analyzed by RT-PCR, and we validated the upregulation of the activating IL-1 ligands *IL1A* and *IL1B* in both cell lines (Figure 3A). Conflicting results were observed with the differential gene expression of *IL1RA* and the remainder of the IL-1 pathway receptors, signaling members, and target genes (Figure 3A–3C) compared to results observed from the microarray gene expression analyses (Table 1). Despite this conflicting gene expression data, we found that there was no difference in the secretion of IL-1α (Figure 3D) and IL-1β (Figure 3E) between ER and ES cells. However, secretion of IL-1RA was significantly downregulated in the ER-cell lines compared to their respective ES-counterparts (Figure 3F). Altogether, these results in Figure 3 suggest that increased IL-1 signaling may be involved in erlotinib resistance and this increased IL-1 signaling in ER-HNSCC cells may be due to reduced IL-1RA protein secretion.

### IL-1 blockade affects IL-6 and IL-8 secretion but not cell viability *in vitro*

**Figure 4:**
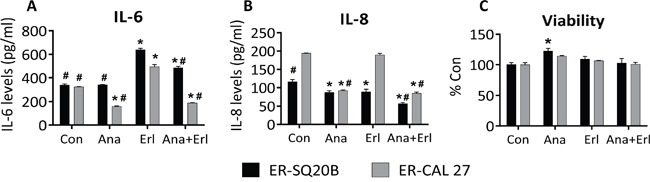
Effect of IL-1 blockade on cytokine secretion and cell viability in erlotinib-resistant (ER) HNSCC cells *in vitro* ER–SQ20B and CAL 27 cells were treated with reagent grade water (Con) or 500 ng/mL anakinra (Ana) in the absence or presence of 5 mM erlotinib (Erl) for 48 h before analyzing for IL-6 **A.** and IL-8 levels **B.** by ELISA and cell viability **C.** N = 6, error bars represent ± standard deviation of the mean. *: p<0.05 versus Con; #: p<0.05 versus Erl.

In order to investigate if IL-1 blockade affects downstream cytokine expression, we pretreated ER-SQ20B and ER-CAL 27 cells with an FDA approved recombinant IL-1 receptor antagonist anakinra for 3-4 hours prior to treatment with erlotinib (5 μM) for 48 h before assessing the secretion of common IL-1 target cytokines such as IL-6 and IL-8. Erlotinib treatment induced a significant increase in the secretion of IL-6 in both ER-cell lines and anakinra significantly reduced this erlotinib-induced IL-6 secretion (Figure 4A). Anakinra significantly suppressed IL-6 secretion as a single agent in ER-CAL 27 cells only (Figure 4A). Anakinra as a single agent and in the presence of erlotinib significantly suppressed IL-8 secretion in both ER-cell lines (Figure 4B). Additionally, erlotinib had no effect on IL-8 secretion in CAL 27 cells but significantly suppressed IL-8 in SQ20B (Figure 4B). Despite the observed effects of IL-1 blockade on cytokine expression (Figure 4A, 4B), there was no change in significant reduction in cell viability *in vitro* after treatment of both ER-cell lines with anakinra alone or in combination with erlotinib compared to control (Figure 4C) suggesting that IL-1 blockade has no effect on erlotinib resistance in HNSCC cell *in vitro*.

### IL-1 blockade overcomes erlotinib resistance *in vivo*

**Figure 5:**
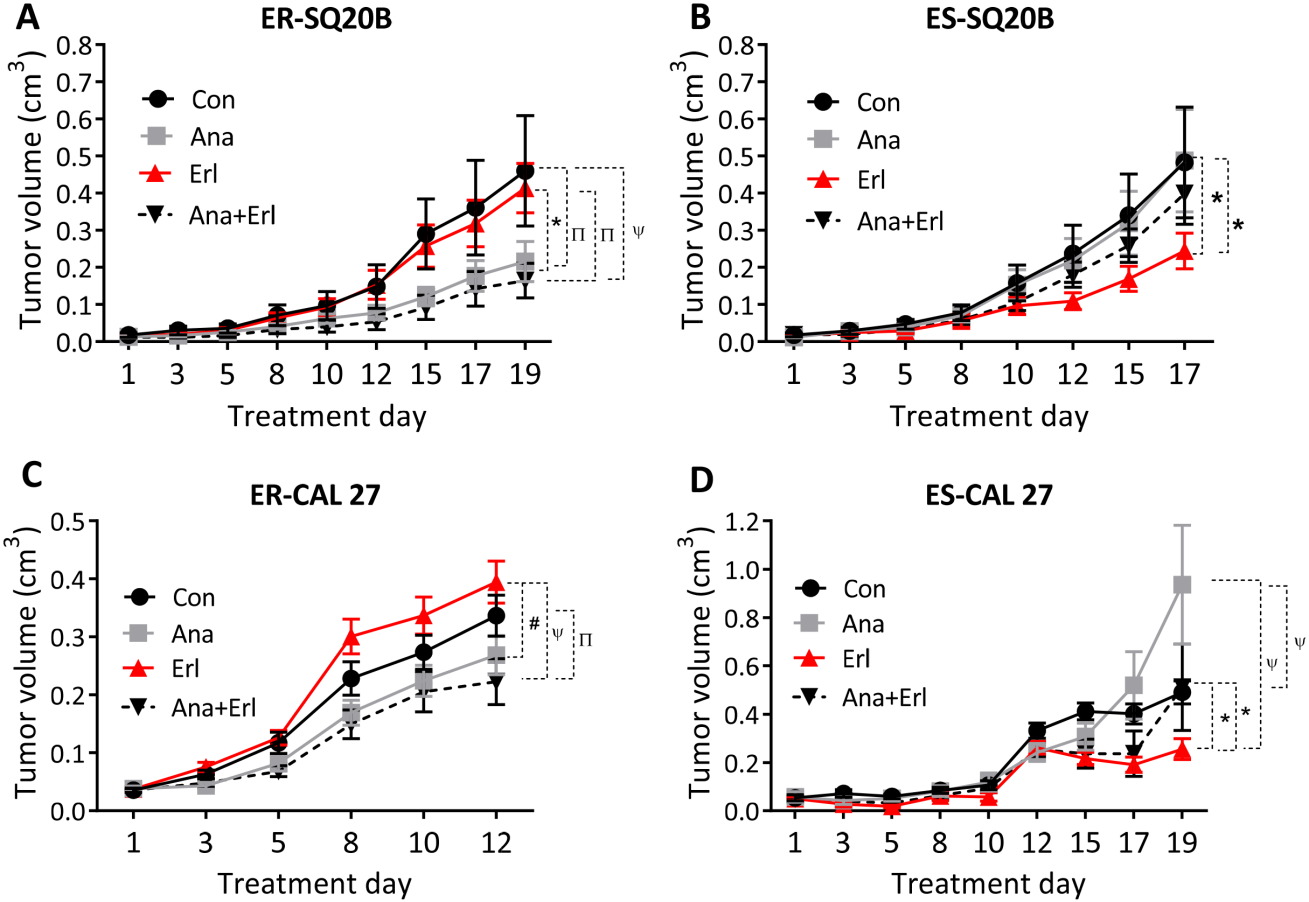
Effect of anakinra on the anti-tumor efficacy of erlotinib in erlotinib resistant (ER) and erlotinib-sensitive (ES) HNSCC cells *in vivo* ER-SQ20B **A.**, ES-SQ20B **B.**, ER-CAL 27 **C.** and ES-CAL 27 **D.** tumor bearing nude mice were treated with water (Con), erlotinib (Erl), anakinra (Ana) or erlotinib+anakinra (Ana+Erl) for 2-3 weeks. Tumor volumes were plotted against days since treatment initiation. Tumor growth graphs were interrupted at the time point where the mice (in any of the treatment groups) began to reach euthanasia criteria due to tumor size or ulcerating tumors. N = 6-11 mice per treatment group. Error bars represent ± standard error of the mean. *p<0.05, ^Π^p<0.01, ^#^p<0.001, ^ψ^p<0.0001.

When anakinra ± erlotinib were tested in an ER-SQ20B xenograft mouse model, we observed that tumors treated with anakinra alone or in combination with erlotinib grew significantly slower than those treated with control or erlotinib-treated tumors (Figure 5A) suggesting that anakinra treatment can overcome erlotinib resistance in this ER-cell line. This result is in contrast to ES-SQ20B xenografts, which were only affected by erlotinib treatment (Figure 5B). In the ER-CAL 27 xenograft model, similar results were observed where tumors treated with anakinra alone and in combination with erlotinib grew slower that control or erlotinib-treated tumors (Figure 5C). Unfortunately, anakinra (alone) treatment was not significantly different from control treatment (Figure 5C) which may be due to the poor tumor response to anakinra observed in male mice bearing CAL 27 xenografts (Supplementary Figure 1A) compared to female mice bearing CAL 27 xenografts (Supplementary Figure 1B). Again, these results could not be duplicated in ES-CAL 27 xenografts where erlotinib treatment was the most effective of all the other treatment groups (Figure 5D). There were no observed apparent toxicities (i.e. skin rash, infections, loss of body weight, overall well-being) in the mice treated with anakinra and/or erlotinib. Together, these results suggest that IL-1 blockade using anakinra could overcome erlotinib resistance in HNSCC cells *in vivo* but has no effect on erlotinib efficacy in ES-HNSCC cells.

### The anti-tumor effect of anakinra is associated with a reduction in angiogenesis

**Figure 6:**
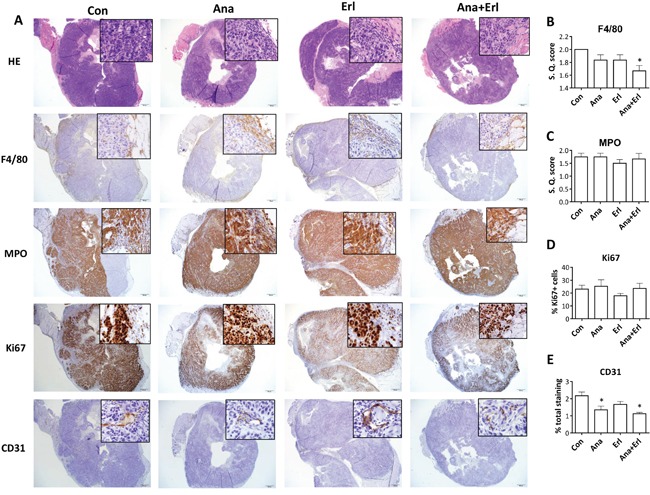
Histopathology and immunostaining of tumor sections from ER-SQ20B xenografts Low (100x) and high (insets; 200x) magnification images were taken of formalin fixed paraffin embedded tumor sections of ER-SQ20B xenografts harvested from mice treated with water (Con), anakinra (Ana), erlotinib (Erl) or anakinra+erlotinib (Ana+Erl) after staining with Hematoxylin and Eosin (H&E), F4/80, myeloperoxidase (MPO), Ki67 and CD31. Semi-quantitative scoring was assigned for F4/80 staining **B.**, and semi-quantitative scoring of peri-tumoral regions was assigned for MPO stained sections **C.** Ki67 staining was quantified using ImageJ software. Percentage Ki67 positive cells are represented by (Ki67-positive cells/total cells in FOV) x 100 **D.** Percentage of CD31 staining was quantified by Aperio slide scanner technology **E.** Each image is a representative of 3 tumor sections per group. All semi-quantitative and quantitative scoring was performed on 3 tumor sections per group. Error bars represent standard error of mean. S.Q. = semi quantitative.

In order to assess the effect of erlotinib and/or anakinra on the tumor microenvironment, tumor tissue was taken from mice bearing ER-SQ20B xenografts shown in Figure 5A for histopathology and immunohistochemistry (IHC) analysis at the end of the drug treatment period. Using H&E staining, we observed no differences in terms of tumor morphology, amount of necrosis, intra-tumor and peri-tumor inflammation (Figure 6A, top row) between the treatment groups. F4/80 staining for murine macrophages was mostly present in the peri-tumor regions of tumor sections (Figure 6A, second row) and only the tumors treated with anakinra+erlotinib had significantly lesser macrophage recruitment compared to that of the control group (Figure 6B). Immunostaining for myeloperoxidase (MPO) showed that both tumor cells and neutrophils (which are mostly in the peri-tumoral region) stained for MPO, making the analysis of neutrophilic recruitment problematic (Figure 6A, third row), however semi-quantitative scoring focusing on the peri-tumoral regions, did not reveal any significant difference among the treatment groups (Figure 6C). Together, these results suggest that the inhibition of tumor growth mediated by anakinra and anakinra+erlotinib (Figure 5A) is likely not mediated by macrophage or neutrophil recruitment in this mouse model. Additionally, there were no significant differences among treatment groups in the proliferation marker Ki67 (Figure 6A, fourth row, 6D) and activated caspase-3 (Supplementary Figure S2) staining. However, CD31 staining for angiogenesis revealed that the tumors treated with anakinra or anakinra+erlotinib had significantly lesser CD31 staining compared to control or erlotinib-treated tumors (Figure 6A, bottom row, 6E). Taken together, these results suggest that anakinra alone or in the presence of erlotinib may inhibit tumor growth in ER-HNSCC tumors by a mechanism involving a reduction in angiogenesis.

### Anakinra modulates circulating levels of proinflammatory cytokines in mice bearing ER-SQ20B xenografts

**Figure 7:**
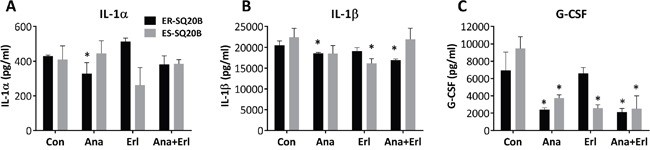
Effect of EGFR and IL-1 blockade on circulating levels of cytokines and chemokines in mice harboring ER- and ES-SQ20B xenografts Sera from mice bearing ER-SQ20B (black bars) and ES-SQ20B (grey bars) xenografts treated with either water (Con), anakinra (Ana), erlotinib (Erl) and anakinra+erlotinib (Ana+Erl) were analyzed for concentrations of IL-α **A.**, IL-1β **B.** and G-CSF **C.** using a mouse Bio-Plex assay. N = 3 mice per treatment group. Error bars represent ± standard error of the mean (SEM). *: p< 0.05 versus Con.

To investigate the effect of anakinra treatment on circulating levels of host proinflammatory cytokines, we measured the levels of 17 cytokines/chemokines from the sera of drug-treated mice harboring ER-SQ20B xenografts (Figure 5A) and ES-SQ20B xenografts (Figure 5B). Mean values of all detectable cytokines and chemokines in all the four treatment groups are shown in Supplementary Table S1. Of the 17 cytokines/chemokines analyzed, only circulating levels of IL-1α, IL-1β, IL-6, G-CSF, IL-12p40 and IL-12p70 were affected by anakinra and/or erlotinib treatment compared to control in mice bearing ER-SQ20B xenografts (Supplementary Table S1) and circulating levels of IL-1β, IL-6, G-CSF, IL-12p40, GM-CSF, IL-33 and MIP-1β were significantly affected in mice bearing ES-SQ20B xenografts (Supplementary Table S1). Notably, treatment with anakinra as a single agent and anakinra+erlotinib (but not erlotinib alone) either significantly suppressed or showed a trend toward the suppression of IL-1α (Figure 7A), IL-1β (Figure 7B) and G-CSF (Figure 7C) in ER-SQ20B tumors compared to control treated tumors. This pattern was not observed in the ES-SQ20B tumors suggesting that the anti-tumor effect of these particular drug treatments observed in mice bearing ER-SQ20B xenografts (Figure 6A) may involve reduced circulating levels of host IL-1α, IL-1β and G-CSF.

### *IL1A* and *IL1RAP* are associated with HNSCC patient survival

**Figure 8:**
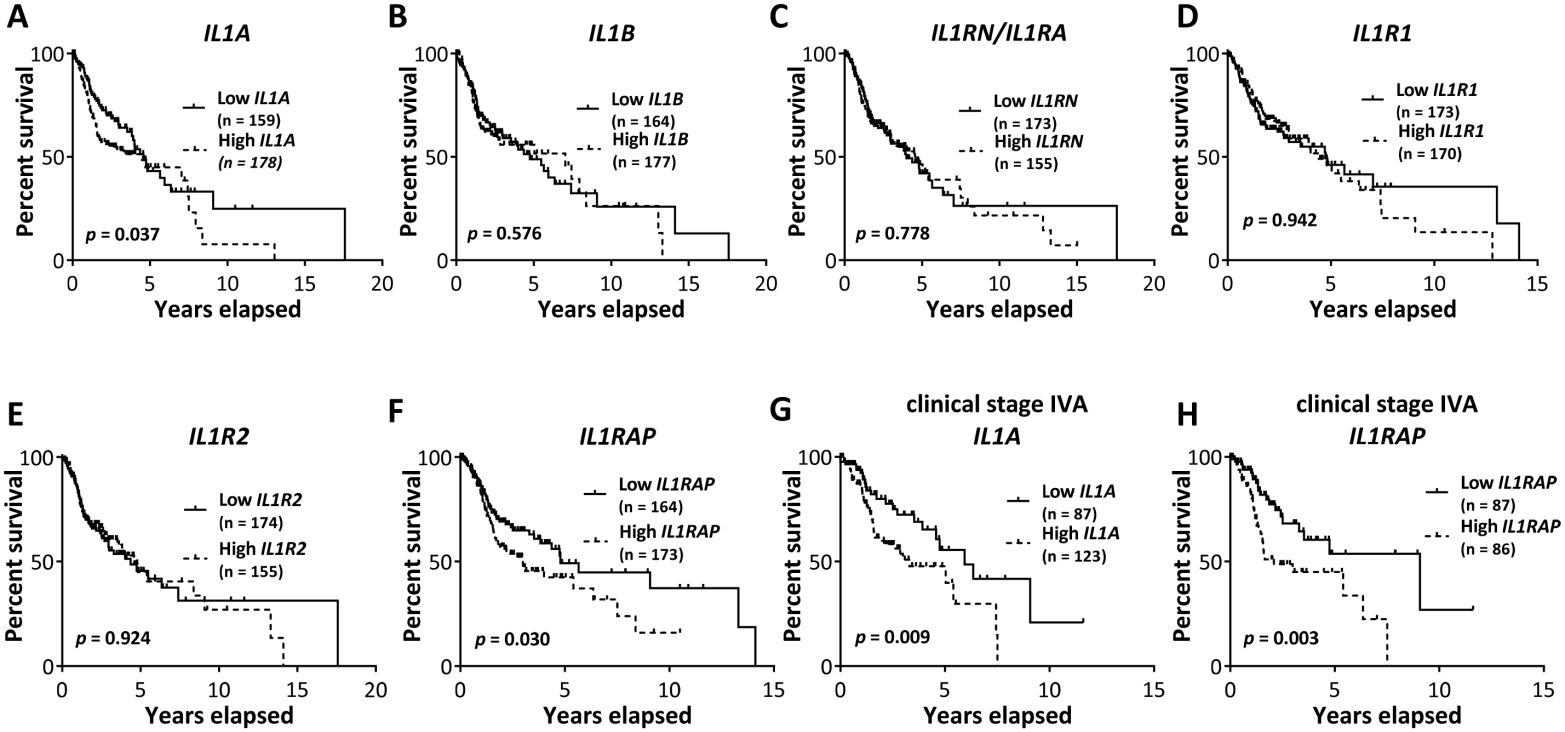
Association of IL-1 pathway gene expression with survival of HNSCC patients Kaplan-Meier survival curves were generated from Pancan normalized HNSCC gene expression data from The Cancer Genome Atlas data portal for HNSCC patients of all clinical stages, sorted into highest and lowest tertile cohorts based on tumor *IL1A* **A.**, *IL1B* **B.**, *IL1RN* **C.**, *IL1R1* **D.**, *IL1R2* **E.** and *IL1RAP* **F.** mRNA expression. Kaplan-Meier survival curves generated from stage IVA HNSCC patients, sorted into highest and lowest tertiles based on tumor *IL1A* **G.** and *IL1RAP* **H.** mRNA expression. Number of subjects in each cohort was indicated in parentheses in respective plots.

To investigate the association between tumor expression of IL-1 pathway ligands and receptors (i.e. *IL1A, IL1B, IL1RN, IL1R1, IL1R2*, and *IL1RAP*) and HNSCC patient survival, we performed Kaplan-Meier survival analyses using gene expression datasets of tumors from HNSCC patients in the TCGA data portal (Figure 8A–8H). Survival analysis on HNSCC patients of all clinical stages (i.e. stage I through IV) revealed that high IL1A gene expression (i.e. highest tertile) was associated with poorer survival compared to those expressing low IL1A gene expression (i.e. lowest tertile) (Figure 8A). Similar results were observed with the IL1RAP gene (Figure 8F). We did not observe any significant differences in survival times among HNSCC patients sorted into highest and lowest tertiles, based on the expression of IL1B, IL1RN, IL1R1, and IL1R2 (Figure 8B–8E). When HNSCC patient were sorted into cohorts of different clinical stages (i.e. stage I, II, III, IVa, IVb, and IVc), we found that high expression of IL1A (Figure 8G) or IL1RAP (Figure 8H) was only significantly associated with poor survival in patients with stage IVa (i.e. moderately advanced) disease. There were not enough HNSCC samples in the TCGA database to perform an appropriate survival analysis on stage IVb or IVc HNSCC patients. Additionally, none of the HNSCC patients analyzed were treated with erlotinib. However, these results suggest that IL1A and IL1RAP gene expression may predict survival in HNSCC patients despite prior treatment regimens, especially in patients with advanced disease.

## DISCUSSION

In this study, we showed that ER-HNSCC cells demonstrate upregulated IL-1 signaling, and blockade of this pathway using anakinra overcame erlotinib resistance *in vivo*. IL-1 is a master cytokine which drives the expression of several inflammatory molecules such as IL-α, IL-1β, IL-6, IL-8 and others involved in tumor growth and spread [17–21]. Previous studies from our lab have determined the contributory roles of IL-α and IL-6 signaling in limiting the efficacy of erlotinib and other EGFR inhibitors in HNSCC [16, 23]. Hence, blocking IL-1 signaling may be a promising strategy to overcome resistance to erlotinib and possibly other EGFR inhibitors in HNSCC.

Blockade of IL-1 signaling by anakinra is an FDA-approved therapy for treating rheumatoid arthritis (RA) and other chronic inflammatory conditions [18]. Interestingly, ‘rheumatic diseases’ and ‘arthritis’ were among the top ten diseases associated with erlotinib resistance in all the four HNSCC cells (Figure 1B) suggesting that drugs used to treat RA and other inflammatory disease may be promising candidates to test in combination with erlotinib for HNSCC therapy. Pathway analysis revealed a significant deregulation of IL-1 signaling pathway in all the four ER-HNSCC cells (Figure 2). When we validated IL-1 signaling pathway by RT-PCR and ELISA, we observed a mismatch between mRNA and protein secretion of IL-1 ligands (i.e. IL-α, IL-1β, and IL-1RA) in ER- *vs.* ES-HNSCC cells (Figure 3A; Figure 3D-3F). It is well documented that mRNA levels do not always correlate with protein expression [24, 25] given the many post-transcriptional (e.g. miRNA-mediated mRNA degradation) and post-translational (e.g. ubiquitinylation) mechanisms that exist to regulate the levels of corresponding protein in a cell [24]. Therefore, if we focus solely on the ELISA results, we observed that ER-HNSCC cells secreted similar levels of IL-α and IL-1β but significantly lower levels of IL-1RA as compared to respective ES-HNSCC cells (Figure 3D–3F). Lower levels of IL-1RA would increase the availability and agonistic activity of IL-1α and IL-1β in ER-HNSCC cells as compared to their ES-HNSCC cells. Hence, IL-1 signaling may be upregulated in ER-HNSCC cells.

In our previous work, we showed that increased IL-1α secretion limited erlotinib efficacy in HNSCC cells [16]. Therefore in these studies, we proposed that erlotinib resistance can be overcome by elevating the levels of IL-1RA using anakinra in ER-HNSCC cells, which would competitively inhibit IL-1 signaling. Exogenous administration of anakinra led to 1: significant reduction in the levels of IL-1 target molecules such as IL-6 and IL-8 in ER-SQ20B and ER-CAL 27 cells *in vitro* (Figure 4A, 4B) and 2: significant growth inhibition of ER-SQ20B and ER-CAL 27 xenografts as compared to their respective controls (Figure 5A, 5C). In support to our hypothesis, these results suggested that anakinra administration overcame erlotinib resistance possibly by inhibiting IL-1 signaling in ER-HNSCC cells. More importantly, lack of growth inhibitory effects of anakinra on ES-HNSCC xenografts (Figure 5B, 5D) unveiled the critical dependency (i.e. addiction) of ER-HNSCC cells on IL-1 signaling for their survival. Since we previously showed that erlotinib induced IL-1α in HNSCC cells [16] and we developed the ER-HNSCC cells by chronically exposing ES-HNSCC cells to erlotinib; it is likely that this chronic exposure had led to sustained activation of IL-1 signaling *via* IL-α release. IL-1 signaling is known to have cell survival effects *via* NF-κB [26]. Therefore, one can reason that sustained exposure of ES-HNSCC cells to erlotinib-induced IL-α may have led to the dependency of HNSCC cells on IL-1 signaling for survival.

The observation that anakinra did not affect cell viability *in vitro* but induced significant tumor growth suppression *in vivo* in ER-SQ20B and ER-CAL 27 suggests that anakinra's mechanism of action likely involves the tumor microenvironment. ER-SQ20B tumors treated with anakinra were significantly less vascularized (as determined by CD31 staining) compared to control or erlotinib treated tumors (Figure 6E). These data support prior findings showing that IL-1RA did not alter tumor cell proliferation rates *in vitro,* but significantly inhibited xenograft growth and neovessel density (as determined by factor VIII staining) in IL-1-producing tumor cell lines [27]. Blockade of IL-1 signaling may have suppressed the secretion of angiogenic molecules such as IL-8 in ER-SQ20B cells similar to our results *in vitro* (Figure 4B) since human IL-8 was shown to interact with mouse chemokine receptor and promote angiogenesis [28–29]. Moreover, circulating levels of angiogenic molecules i.e. IL-1β, G-CSF and to some extent IL-1α were also reduced in the mice that were administered with anakinra (Figure 7A–7C). This may be due to the fact that anakinra can recognize and bind to human and host murine IL-1R1. The ability of anakinra to inhibit the murine IL-1 receptor signaling has been demonstrated in several studies [30–31]. Therefore, we can hypothesize that anakinra administration led to significant suppression of tumor-derived and host-derived/circulating IL-1 target angiogenic molecules which contributed to the reduced tumor-associated angiogenesis and reduced tumor growth.

We have demonstrated that targeting IL-1 signaling pathway by administering anakinra is a new and promising approach to overcome the problem of erlotinib resistance in HNSCC. Anakinra as a single agent was shown to suppress tumor growth of IL-1-producing, but not non-IL-1-producing, tumor cell lines [27] and anakinra has been shown to be effective in combination with other anti-tumor agents in various other disease models [32–35]. Large numbers of patients with chronic inflammatory diseases have safely been using anakinra for a long period of time since its approval by FDA. There are quite a few ongoing clinical trials (n = 9; clinicaltrials.gov) testing the efficacy of anakinra in different cancers. Moreover, downregulation of the IL-1 pathway using the IL-1ra was shown to alleviate cancer-associated cachexia [36–39] and EGFR inhibitor-induced skin toxicity [40–42]. Given these benefits, combining anakinra with erlotinib or other EGFR inhibitors should be strongly considered as administering anakinra may not only overcome erlotinib resistance but also improve overall wellbeing in HNSCC patients.

## MATERIALS AND METHODS

### Cell lines and culture

FaDu, CAL 27, and SCC-25 cell lines were obtained from American Type Culture Collection (ATCC, Manassas, VA). The SQ20B cell line [43] was gifted by Dr. Anjali Gupta (Department of Radiation Oncology, The University of Iowa). All four HNSCC cell lines express EGFR and are sensitive to EGFR inhibitors. Erlotinib-resistant HNSCC cell lines were developed as described previously [22]. FaDu, CAL 27, and SQ20B were cultured in Dulbecco's Modified Eagle's Medium (DMEM) containing 4 mM L-glutamine, 1 mM sodium pyruvate, 1.5 g/L sodium bicarbonate and 4.5 g/L glucose with 10% Fetal Bovine Serum (FBS; Hyclone, Logan, UT). SCC-25 cells were cultured in a 1:1 mixture of DMEM and Ham's F12 medium containing 1.2 g/L sodium bicarbonate, 2.5 mM L-glutamine, 15 mM HEPES, 0.5 mM sodium pyruvate, 4.5 g/L glucose, and 400 ng/mL hydrocortisone with 10% FBS. Cell cultures were maintained in a humidified atmosphere at 37°C and 5% CO_2_. Erlotinib-resistant cell lines were maintained in 5 μM erlotinib-containing culture media and were maintained in erlotinib-free media for at least a week before they were used for experiments. All HNSCC cell lines are EGFR positive and are sensitive to EGFR inhibitors. All cell lines were authenticated by the ATCC for viability (before freezing and after thawing), growth, morphology and isoenzymology. Cells were stored according to the supplier's instructions and used over a course of no more than 3 months after resuscitation of frozen aliquots. Cultures were maintained in 5% CO_2_ and air humidified in a 37°C incubator.

### Drugs

Erlotinib (Tarceva® for use in *in vivo* experiments; Cayman chemical, MI, USA for *in vitro* experiments), and anakinra (Kineret®) were obtained from the inpatient pharmacy at the University of Iowa Hospitals and Clinics. Dimethyl sulfoxide (DMSO) and ultrapure water were used as controls and obtained from Merck (Billerica, Massachusetts, USA). Erlotinib was dissolved in DMSO for *in vitro* experiments or suspended in water for *in vivo* experiments. Anakinra was diluted in ultrapure water for *in vitro* and *in vivo* experiments. Diluted drugs were added directly to cell culture in order to achieve the specified drug concentrations.

### Cell viability assay

HNSCC cells were seeded in 96-well plate (5 × 10^3^ cells/100 μl media/well) and incubated overnight under standard cell culture conditions before adding 100 μl of indicated drugs for 48 h. At the end of incubation, live cell cultures were incubated with PrestoBlue™ cell viability reagent (Invitrogen, USA) for 30 min at 37°C before measuring cell viability according to the manufacturer's protocol.

### Gene expression profiling

Gene expression profiling was performed on all erlotinib-sensitive (ES) and erlotinib-resistant (ER) FaDu, CAL 27, SCC-25 and SQ20B HNSCC cell lines (n=8) at the Iowa Institute of Human Genomics (IIHG; The University of Iowa, Iowa City) as described previously [22]. The complete raw microarray datasets have been deposited in NCBI's Gene Expression Omnibus and are accessible through GEO Series accession number GSE62061 (https://www.ncbi.nlm.nih.gov/geo/query/acc.cgi?acc=GSE62061).

### Enrichment analysis

Log-transformed and quantile normalized gene expression data was subjected to one-way ANOVA with multiple test corrections using Partek Genomics Suite 6.5 (Partek®). Differential gene expression data was uploaded to MetaCore™ GeneGo software (https://portal.genego.com/) for enrichment analysis which includes ‘Gene Ontology Processes’, ‘Diseases (by biomarkers)’, and ‘pathway analysis’. Threshold values of ± 2 and p-value of 0.05 was used as significance criteria for enrichment analysis.

### Reverse transcription PCR

RNA isolation and RT-PCR was performed as described previously [33]. Briefly, HNSCC cells were seeded in 60 mm dishes (2 × 10^5^ cells/dish) and incubated for 48 h in 5 μM DMSO before isolating total RNA using RNeasy Plus mini kit (QIAGEN) as per manufacturer's protocol. 2 μg of RNA was reverse transcribed to cDNA using iScript cDNA synthesis kit (Bio-Rad). The resultant cDNA samples were used to perform quantitative PCR analysis. Oligonucleotide primers were obtained from Integrated DNA Technologies (IDT; Coralville, IA) and presented in Supplementary Table S2. PCR data was presented in fold change values. Fold change values were calculated by doing the anti-log of delta delta CT values (i.e. 2^-delta delta CT values). Each assay was performed three times and results were presented as mean ± standard error of mean.

### ELISA

Cell culture supernatants were harvested and centrifuged at 14,000 × g for 15 min at 4°C to remove cellular debris. Concentrations of secreted IL-1α, IL-1β, IL-1Ra, IL-6, and IL-8 in the supernatants of indicated experimental conditions were detected using Human Duoset or Quantikine ELISA kits (R & D Systems, Minneapolis, MN) as per manufacturer's protocol. Concentrations of cytokines were normalized to number of viable cells.

### Xenograft experiments

Male and female athymic *nu/nu* mice (4–6 weeks old) were purchased from Harlan Laboratories (Indianapolis, IN). Mice were housed in a pathogen-free barrier room in the Animal Care Facility at the University of Iowa and handled using aseptic procedures. All procedures were approved by the IACUC committee of the University of Iowa and conformed to the guidelines established by the NIH. Mice were allowed at least 4 days to acclimate prior to beginning experimentation, and were given ad libitum access to food and water. Tumor cells were inoculated into nude mice by subcutaneous injection of 0.1 mL aliquots of saline containing 1 × 10^6^ HNSCC cells as indicated into the right flank using 26-gauge needles (BD PrecisionGlide™ Needles, BD, New Jersey). Mice started drug treatment at an average tumor volume of 0.01 cm^3^ (for SQ20B) or 0.03 cm^3^ (for CAL 27). Mice were evaluated daily and tumor measurements taken three times per week using Vernier calipers. Tumor volumes were calculated using the formula for an oblong sphere: volume = (width^2^ × length), where the length was the longest dimension, and width was the dimension perpendicular to length.

### *In vivo* drug administration

Mice were divided into 4 groups; Control group: 100 μL of ultrapure water intraperitoneal injection 5 times per week; Erlotinib group: 100 μL of erlotinib (12.5 mg/kg) administered orally 5 times per week. Anakinra group: 100 μL anakinra (10 mg/kg) intraperitoneal injection 7 times per week. Anakinra+erlotinib group: received anakinra and erlotinib as mentioned above. Treatment period was 3 weeks for all cell lines. Tumor growth graphs were interrupted at the time point where the mice (in any of the treatment groups) began to reach euthanasia criteria due to tumor size or ulcerating tumors. Mice were euthanized via CO_2_ gas asphyxiation when tumor diameter exceeded 15 mm in any dimension.

### Tumor histopathology and immunohistochemistry

Formalin fixed paraffin embedded tissues were routinely stained with hematoxylin and eosin (H&E) and immunohistochemistry (IHC) was performed for F4/80, CD31, Ki67 and activated caspase-3. Antigen unmasking of paraffin sections was performed (citrate buffer, pH 6) in a decloaker for activated caspase-3 and Ki67. For CD31 and F4/80 antigen unmasking was performed using Proteinase K for 10 minutes. Endogenous peroxidase activity was quenched with 3% hydrogen peroxide and either 1.5% horse serum (CD31), 10% goat serum (activated caspase-3) or Background Buster Innovex Company) (Ki67 and F4/80) were used to block non-specific staining. Sections were incubated with monoclonal rat anti-F4/80 (Serotec MCAP497) at 1:200, monoclonal rat anti-CD31 (BD Pharmingen 550274) at 1:200, monoclonal rabbit anti-Ki67 (Abcam ab137876) at 1:400, or rabbit polyclonal activated caspase-3 (Cell Signaling Company #9661) for 1 hour. Slides were then incubated with the appropriate secondary antibody and detection (DAKO Rabbit Envision HRP System reagent for 30 minutes for Ki67, biotinylated anti-rabbit IgG at 1:500 followed by ABC for cleaved caspase-3, Biocare Medical Polymer Kit for F4/80 or biotinylated anti-rat IgG at 1:200 followed by ABC for CD31). Slides were then developed with DAKO DAB plus for 5 minutes followed by DAB Enhancer for 3 minutes before being counterstained with Hematoxylin. Digital image analysis was performed using a color deconvolution algorithm on Aperio slide scanning technology and software. Cells which took the pertinent stain were defined as positive cells. For Ki67 staining, percentage Ki67 positive cells were represented by (Ki67-positive cells/ total cells in FOV) x 100. All tumors were histopathologically examined by a board-certified veterinary pathologist. Three different tumor samples from each treatment group were assessed.

### Bio-Plex assay

Whole blood samples were collected from the abdominal aorta and allowed to clot at room temperature for 30 – 45 min. Clotted blood samples were centrifuged at 1,000 x g for 15 min at 4°C and the sera were transferred to separate tubes. To completely remove platelets and precipitates, serum samples were centrifuged again at 10,000 x g for 10 min at 4°C. All samples were stored at −70°C before analysis. The concentrations of proinflammatory analytes (i.e. cytokines and chemokines) in the mouse sera were determined using a mouse Bio-Plex 17 panel assay, as per the manufacturer's instructions (Bio-Plex Pro mouse cytokine Group 17-Plex Panel, Bio-Rad Hercules, California, USA). The list of cytokines and chemokines analyzed along with their detection range (in pg/ml) are presented in Supplementary Table S3. Briefly, serum samples were diluted 1:4 in sample diluent and incubated for 60 min (850 rpm agitation, at room temperature) in the dark with capture antibody-coupled magnetic beads. The samples were washed three times in a Bio-Plex Pro wash station before incubating them for 30 min (850 rpm agitation, room temperature) in the dark with biotinylated detection antibody. Then streptavidin-phycoerythrin was added to detect each captured analyte. Fluorescent signals were quantified using a Bio-Plex array reader. Bio-Plex Manager software was used to calculate analyte concentrations.

### TCGA analysis

Publicly available, level_3, log2(x+1) transformed, pancan normalized Head and Neck Squamous Cell Carcinoma gene expression (by RNAseq) data from The Cancer Genome Atlas (TCGA) data portal was used to analyze the association between the expression of different IL-1 pathway genes and patient survival. The data were downloaded through the University of California, Santa Cruz Cancer (UCSC) Browser. For survival analysis, primary tumor data was sorted based on the expression levels of different IL-1 pathway genes (such as *IL1A*, *IL1B*, *IL1RN*, *IL1R1*, *IL1R2*, *IL1RAP*). The highest and lowest tertiles were used for this analysis. A Kaplan-Meier plot (GraphPad Prism 6, GraphPad Software, San Diego, CA, USA) was used to analyze survival of patients expressing different (high = highest tertile; low = lowest tertile) levels of IL-1 pathway genes in their primary tumor tissues.

### Statistical analysis

Statistical analysis of significant differences was performed using GraphPad Prism 6 software. Multiple t-tests with false discovery rate (FDR) < 0.05 was performed to test the significant differences in gene expressions (by RT-PCR) of IL-1 pathway between ES- and ER- HNSCC cells *in vitro*. One-way ANOVA with Tukey's post-test was performed to test the differences of means among different treatment groups in the *in vitro* experiments. One-way ANOVA (with Holm-Sidak post-test) was performed to compare the mean of each treatment group with that of control group to analyze IHC and bio-plex data. Two-way ANOVA with Tukey's post-test was used to estimate and compare the group-specific change in tumor growth curves. Kaplan-Meier survival plots were analyzed with the Log-rank (Mantel-Cox) test using the GraphPad Prism 6 software. A probability (P) value of 0.05 or lower was considered significant.

## SUPPLEMENTARY FIGURES AND TABLES


