# Supplementary material for: Interleukin-1 blockade overcomes erlotinib resistance in head and neck squamous cell carcinoma
Source: Oncotarget. 2016 Oct 12;7(46):76087–100. doi: 10.18632/oncotarget.12590 (PMC5342798; doi:10.18632/oncotarget.12590)
Supplement: Supplementary file 1 [file oncotarget-07-76087-s001.pdf]

Interleukin-1 blockade overcomes erlotinib resistance in head and neck squamous cell carcinoma

SUPPLEMENTARY FIGURES AND TABLES

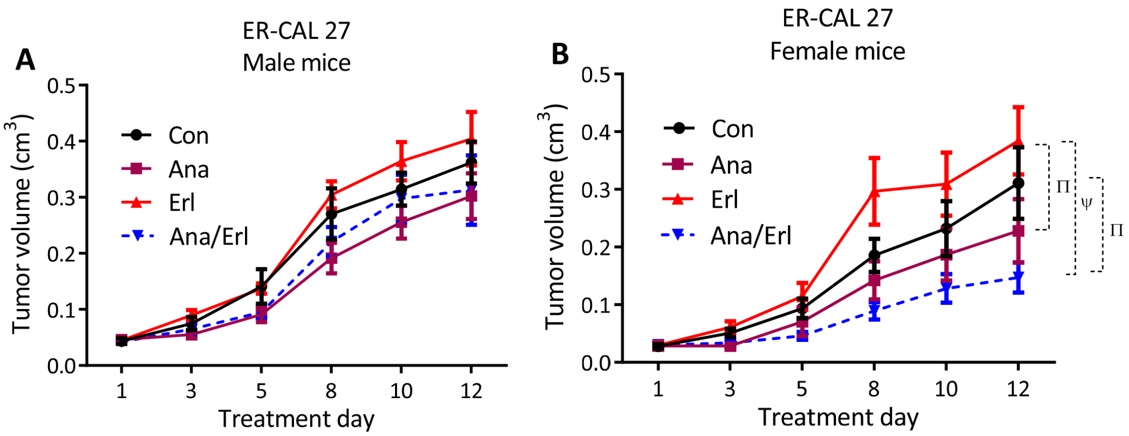

Supplementary Figure S1: Effect of IL-1 blockade on growth of erlotinib-resistant CAL 27 xenografts. Erlotinib-resistant CAL 27 tumor bearing male **A.** and female **B.** mice were treated with reagent grade water (Con), erlotinib (Erl), anakinra (Ana) or erlotinib+anakinra (Ana+Erl) for 2 weeks. Tumor volumes were plotted against days since treatment initiation. Tumor growth graphs were interrupted at the time point where the mice (in any of the treatment groups) began to reach euthanasia criteria due to tumor size or ulcerating tumors. N = 5-6 mice per treatment group. Error bars represent ± SEM. $^{\Pi}p<0.01$, $^{\psi}p<0.0001$.

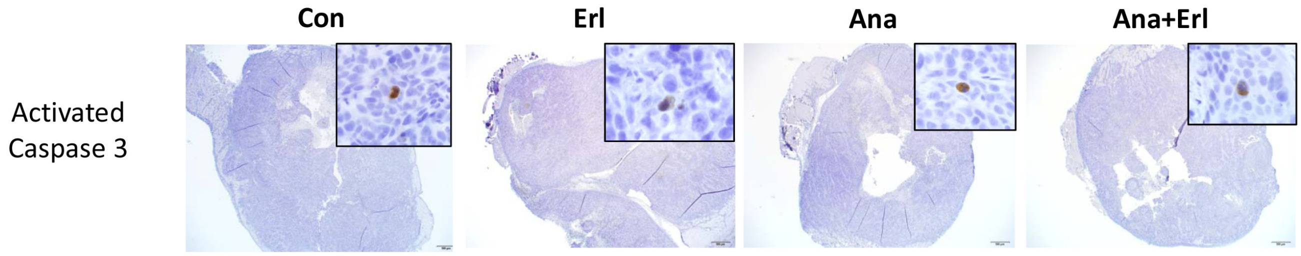

Supplementary Figure S2: Caspase 3 immunostaining of tumor sections from ER-SQ20B xenografts. Images represent low (100x) and high (insets; 200x) magnification images of (formalin fixed paraffin embedded) tumor sections of ER-SQ20B xenografts harvested from mice treated with either reagent grade water (Con), erlotinib (Erl), anakinra (Ana) or erlotinib+anakinra (Ana+Erl), and immunostained with caspase 3 **A-D.** Each image is a representative of 3 tumor sections per group.

Supplementary Table S1: Mean values of cytokines and chemokines in mouse sera harboring ER-SQ20B and ES-SQ20B xenografts

S. No	Analyte name	ER-SQ20B				ES-SQ20B			
		Con	Ana	Erl	Ana/Erl	Con	Ana	Erl	Ana/Erl
1	IL-1α	429.04	328.04	513.49	381.77	409.43	445.00	262.05	384.62
2	IL-1β	20470.97	18599.33	19062.88	16929.72	22369.26	18520.77	16142.87	21874.55
3	IL-6	783.90	1641.4	881.01	496.73	3086.17	559.51	407.51	603.90
4	IL-10	1537.49	2361.26	1476.59	1585.65	1920.27	1159.17	1216.61	1615.36
5	IL-12p40	775.87	1558.19	976.03	662.54	1305.01	1100.22	1607.7	827.52
6	IL-12p70	16036.21	25816.43	21093.08	21909.25	24790.03	18487.5	21408.9	24299.67
7	IL-17A	8352.79	9872.44	8316.59	10998.64	9363.22	6042.03	10719.24	4829.17
8	G-CSF	6927.53	2388.51	6583.7	2109.24	9470.65	3730.97	2562.17	2503.73
9	GM-CSF	3609.79	3438.63	3492.28	3289.25	4019	3060.90	2976.45	3232.22
10	IFN-γ	3288.32	3719.84	3633.49	3654.58	3806.40	3181.04	3129.58	3591.68
11	KC	2991.46	3787.78	3239.73	2145.72	7824.58	1857.37	1871.22	1734.9
12	MCP-1	18006.38	18647.32	17892.85	17041.33	18475.53	15825.53	14227.5	16986.81
13	MIP-1α	1138.05	1206.23	1146.29	972.68	1305.63	919.79	899.80	1153.06
14	MIP-1β	2884.65	2837.63	2954.01	2712.24	3160.27	2575.14	2146.06	2641.29
15	TNF-α	30261.16	32736.19	32810.85	32997.13	34757.54	29676.18	24845.2	32143.51
16	IL-23p19	ND	ND	ND	ND	ND	ND	ND	ND
17	IL-33	228.07	273.79	241.18	260.67	297.75	250.65	247.74	255.41

Note: Con = Control; Ana = Anakinra; Erl = Erlotinib; Ana/Erl = Anakinra + Erlotinib. ND = not detectable.

Supplementary Table S2: List of primers for RT-PCR

Gene	Forward primer (5'-> 3')	Reverse primer (5'-> 3')
IL1A	CCGTGAGTTTCCCAGAAGAA	ACTGCCCAAGATGAAGACCA
IL1B	AAGCCCTTGCTGTAGTGGTG	GAAGCTGATGGCCCTAAACA
IL1RA	GCTTGCATCTTGCTGGATTT	CCTCAGAAGACCTCCTGTCCT
IL1R1	TTGGGTTAAGAGGACAGGGA	TGATTTCTTCTCTGGAGGCTG
IL1R2	ACAACTTCCAGAGGACACGG	CCTGCTGGAGGTGAAAGTCT
IL1RAP	ATTAAAGGGAGGGGCAAGAG	CCTCTCAGCTTCCCAAGAAA
MYD88	TCCTGCTGCTGCTTCAAGAT	GACTGCTCGAGCTGCTTACC
TOLLIP	CGGGAGCTCACCGATGT	GTCACTGTCGGGTCGGC
IRAK4	TAGTCCAACATTGAGGCAGC	GTTCTTCTGTCGCCGGCTT
IRAK1	CTTCTCAAAGCCACTCCAGC	GAGACCTTGGCTGGTCAGAG
TRAF6	GCCACACAGCAGTCACTTTC	TCCCCGCGCACTAGAAC
CXCL8	AAATTTGGGGTGGAAAGGTT	TCCTGATTTCTGCAGCTCTGT
CCL2	AGGTGACTGGGGCATTGAT	GCCTCCAGCATGAAAGTCTC
PTGS2	CCGGGTACAATCGCACTTAT	GGCGCTCAGCCATACAG
NFKBIA	AAAGCCAGGTCTCCCTTCAC	CAGCAGCTCACCGAGGAC
DUSP1	CAGTGGACAAACACCCTTCC	AGGACAACCACAAGGCAGAC
GAPDH	AATGAAGGGG TCATTGATGG	AAGGTGAAGGTCGGAGTCAA
18S	CCTTGGATGTGGTAGC CGTTT	AACTTTCGATGGTAGTCGCCG

Supplementary Table S3: List of cytokines and chemokines analyzed by Bio-Plex assay

S. No	Analyte name	lower limit (pg/ml)	higher limit (pg/ml)
1	IL-1α	1.28	20970
2	IL-1β	3.82	62650
3	IL-6	1.27	20832
4	IL-10	1.2	19679
5	IL-12p40	1.58	25894
6	IL-12p70	3.2	52439
7	IL-17A	2.73	44706
8	G-CSF	4.73	77459
9	GM-CSF	1.58	25858
10	IFN-γ	0.81	13205
11	KC	2.01	32958
12	MCP-1	1.97	32200
13	MIP-1α	0.89	14653
14	MIP-1β	0.62	10197
15	TNF-α	3.36	55115
16	IL-23p19	7.85	128603
17	IL-33	1.11	18119
